# Draft Genome Sequence of *Salipaludibacillus keqinensis* ACCC 60430^T^, an Aerobic Halophilic Bacterium Isolated from a Salt Lake

**DOI:** 10.1128/MRA.00339-19

**Published:** 2019-06-06

**Authors:** Luna Dong, Shuangyan Wang, Hao Cao, Baisuo Zhao, Kun Wu, Haisheng Wang

**Affiliations:** a Graduate School, Chinese Academy of Agricultural Sciences, Beijing, People’s Republic of China; b College of Life Science, Henan Agricultural University, Zhengzhou, People’s Republic of China; Georgia Institute of Technology

## Abstract

The haloalkaliphilic bacterium Salipaludibacillus keqinensis ACCC 60430^T^, which grows optimally at 8.0% (wt/vol) Na^+^ and pH 9.0, was isolated from Keqin Lake in Qiqihaer, China. The draft genome includes 4,006 predicted genes and 3,784 coding sequences (CDSs). Genomic analysis showed that various genes may explain the mechanism of salt and alkali resistance.

## Abstract (precis)

The haloalkaliphilic bacterium Salipaludibacillus keqinensis ACCC 60430^T^, which grows optimally at 8.0% (wt/vol) Na^+^ and pH 9.0, was isolated from Keqin Lake in Qiqihaer, China. The draft genome includes 4,006 predicted genes and 3,784 coding sequences (CDSs).

## ANNOUNCEMENT

*Salipaludibacillus keqinensis* ACCC 60430^T^ is a strictly aerobic Gram-positive bacterium isolated using serial dilutions from Keqin Lake in Heilongjiang Province, China, and characterized and described as *Salipaludibacillus keqinensis* by Wang et al. (1). Growth is observed at 4 to 40°C (optimum, 25°C), at pH 8.0 to 10.0 (optimum, 9.0), and with up to 16.0% (wt/vol) NaCl (optimum, 8.0%) (1). To understand the resistance mechanism for survival under saline-alkaline conditions, genome sequencing of *S. keqinensis* ACCC 60430^T^ was performed using an Illumina HiSeq 4000 sequencer.

Cells of *S. keqinensis* ACCC 60430^T^ grown on LB medium at pH 9.0 and 25°C with 8% (wt/vol) NaCl for 48 h were collected (1), and genomic DNA was extracted using a microbial DNA isolation kit (Aidlab Biotech, Beijing, China) according to the manufacturer’s instructions. The genome sequencing library was constructed using the TruSeq DNA Nano library prep kit for Illumina. Sequencing was performed by the Illumina HiSeq 4000 sequencer with a paired-end read length of 2 × 150 bp at approximately 240× coverage. The reads were quality inspected with the default program parameters using FastQC v0.11.8 and Quake (2). Genomic sequence contigs were *de novo* assembled using the MicrobeTrakr Plus v0.9.1 (Zeta Biosciences, Shanghai, China). BWA-MEM v0.7.12 (3) was used post-assembly sequence correction according to the manufacturer’s instructions. Automatic annotations were performed using the NCBI Prokaryotic Genome Annotation Pipeline (PGAP) (4) (https://www.ncbi.nlm.nih.gov/genome/annotation_prok/), and checked by the GenBank curation team for the standard requirements. A total of 4,805,090 reads were assembled into 15 contigs, with a total length of 4,150,426 bp, G+C contents of 39.6%, and an *N_50_* value of 1,053,560 bp. Among the 4,006 genes predicted, 3,874 were potential protein-coding genes (coding sequences [CDSs]). We also identified 92 RNAs, including 12 rRNAs (8 5S RNAs, 3 16S RNAs, and 1 23S RNA), 75 tRNAs, and 5 noncoding RNAs (ncRNAs).

Genome sequence analysis of *S. keqinensis* ACCC 60430^T^ revealed its life characteristics of high salt and alkali resistance. The genome includes 1 gene cluster (*betL betP betS betT betT1 betT*) responsible for glycine betaine synthesis from choline and 6 *ectT* genes responsible for ectoine biosynthesis from aspartate semialdehyde. Eight genes encode glycine betaine-binding protein OpuAC, and 9 genes encode glycine betaine/proline betaine-binding periplasmic protein. All of them are very effective osmotic pressure regulators (5, 6). Furthermore, the presences of 5 genes (3 TrkA type and 2 TrkH type) are responsible for K^+^ uptake systems, implying that *S. keqinensis* ACCC 60430^T^ can use K^+^ as an osmolyte to obtain isosmotic cytoplasm by a salt-in strategy when coping with a rapid osmotic shock (7). Alkaliphilic *S. keqinensis* ACCC 60430^T^ has 6 genes encoding Na^+^/H^+^ antiporters (8), 1 *asp23* gene encoding alkaline shock protein 23, and 2 genes encoding Ca^2+^/H^+^ antiporter (9), which may enable *S. keqinensis* ACCC 60430^T^ survival in alkaline environments (10). These predicted genes likely play a role in the salt and alkaline tolerance of *S. keqinensis* ACCC 60430^T^.

### Data availability.

The draft genome assembly of *S. keqinensis* ACCC 60430^T^ has been deposited at DDBJ/ENA/GenBank under the accession number PDOD00000000. Raw sequencing reads have been submitted to the Sequence Read Archive (SRA accession number SRR8759055).
